# Anthracnose Controlled by Essential Oils: Are Nanoemulsion-Based Films and Coatings a Viable and Efficient Technology for Tropical Fruit Preservation?

**DOI:** 10.3390/foods12020279

**Published:** 2023-01-06

**Authors:** Tamires Sousa de Oliveira, André Mesquita Magalhães Costa, Lourdes Maria Corrêa Cabral, Otniel Freitas-Silva, Amauri Rosenthal, Renata Valeriano Tonon

**Affiliations:** 1 Programa de Pós-Graduação em Ciência de Alimentos, Instituto de Química, Universidade Federal do Rio de Janeiro, Rio de Janeiro 21941-909, RJ, Brazil; 2 Embrapa Agroindústria de Alimentos, Av. das Américas, 29501, Rio de Janeiro 23020-470, RJ, Brazil

**Keywords:** anthracnose, *Colletotrichum*, essential oils, nanoemulsions, films, coatings

## Abstract

Post-harvest diseases can be a huge problem for the tropical fruit sector. These fruits are generally consumed in natura; thus, their integrity and appearance directly affect commercialization and consumer desire. Anthracnose is caused by fungi of the genus *Colletotrichum* and affects tropical fruits, resulting in lesions that impair their appearance and consumption. Antifungals generally used to treat anthracnose can be harmful to human health, as well as to the environment. Therefore, essential oils (EO) have been investigated as natural biofungicides, successfully controlling anthracnose symptoms. The hydrophobicity, high volatility, and oxidative instability of essential oils limit their direct application; hence, these oils must be stabilized before food application. Distinct delivery systems have already been proposed to protect/stabilize EOs, and nanotechnology has recently reshaped the food application limits of EOs. This review presents robust data regarding nanotechnology application and EO antifungal properties, providing new perspectives to further improve the results already achieved in the treatment of anthracnose. Additionally, it evaluates the current scenario involving the application of EO directly or incorporated in films and coatings for anthracnose treatment in tropical fruits, which is of great importance, especially for those fruits intended for exportation that may have a prolonged shelf life.

## 1. Introduction

Anthracnose is a serious post-harvest disease that affects various tropical and subtropical fruits such as banana, mango, papaya, and avocado [1]. The infection is caused by fungi of the genus *Colletotrichum*, mainly in the flowering stage of the fruits, remaining latent until development after harvest and extending during storage. Infection can cause an undesirable visual appearance, accelerate fruit ripening, and, in more advanced stages, lead to fruit rot. These symptoms impair the commercialization and exportation of fruits, restraining selling and consumption [2,3].

In addition to the economic and fruit impacts, the *Colletotrichum* genus may pose a risk to human health, because the species *C. atramentum*, *C. graminicola*, *C. dematium*, and *C. gloeosporioides* have been reported to cause keratomicosis. *C. truncatum* was erroneously associated with five cases of ophthalmic infections originally caused by *C. dematium* [4]. This study revealed, at the time, the importance of using molecular methods to identify species. Recently, the species *C. chlorophyti* was found to be responsible for mycotic keratitis in an 82-year-old man [5].

Synthetic fungicides such as thiabendazole and imazalil are the main treatments used in the management of fruits to minimize the effects of infection [1,3]. However, several phytopathogens are resistant to thiabendazole. Fungicides can be harmful to human health, as accumulation in tissues can generate hepatotoxicity, adrenal gland toxicity, carcinogenicity/mutagenicity, nephrotoxicity, and metabolic disorder. In addition, they can cause damage to the environment and affect biodiversity. From the food market point of view, consumers are becoming averse to the presence of synthetic additives. Thus, it is necessary to search for effective, safe, and natural treatments that do not impact consumers’ health or the environment and, when possible, promote no change in the sensory properties of foods [6,7].

In this scenario, natural fungicides are being applied as a tool for microbiological control and, therefore, for post-harvest diseases. The use of essential oils to prevent the spread of infection has been studied as a natural fungicide option. Andrade and Vieira [8], for example, studied the effect of anise (*Pimpinella anisum*), tea tree (*Melaleuca alternifolia*), lemongrass (*Cymbopogon citratus*), mint (*Mentha piperita*), rosemary (*Rosmarinus officinalis*), and cinnamon (*Cinnamomum zeylanicum*) essential oils through in vivo (direct application) and in vitro tests to assess conidia germination. The authors noticed a fungitoxic and fungistatic effect of most oils used in different concentrations. Another study demonstrated through in vitro tests the potential use of cinnamon essential oil (*Cinnamomum zeylanicum* or *Cinnamomum verum*) on mycelial growth and spore twinning of *C. acutatum* isolated from kiwi. In this study, cinnamon oil showed fungistatic and fungicidal effects at concentrations of 0.175 µL/mL and 0.2 µL/mL, respectively [9].

The direct application of EOs to the fruit surface becomes a challenge, due to the volatility of EOs [10]. Hence, it is common to apply essential oils in formulations of polymeric coatings for fruits, aiming to increase their shelf life. However, the high hydrophobicity of these substances makes it difficult to achieve a homogeneous dispersion and spreadability over the whole fruit surface when it is simply added to the coating formulation.

A delivery system designed to protect and deliver the desired compounds at the right time and place is a viable alternative to overcome the challenges of using essential oils in fruits. In this sense, the application of EOs in the form of emulsions is a strategy to improve their dispersibility and homogeneity. Emulsions are systems in which two immiscible liquids are homogenized to form a mixture of spherical droplets (dispersed phase) in a surrounding liquid (continuous phase). The essential oils are usually incorporated into coatings in the form of oil-in-water (o/w) emulsions. Depending on the concentration, conventional EO emulsions can generate changes in fruit color and flavor, in addition to leading to EO degradation when exposed to extreme environmental conditions (temperature, pH, oxygen, light, and moisture) [11]. Another drawback is the fast release of volatile compounds, which can impair their biological action on fruit preservation [12].

When dealing with essential oils for coating or film formulation, nanoemulsions have received substantial attention in recent years due to their functional and physicochemical properties. Nanoemulsions are emulsions with a droplet radius ranging between 10 and 100 nm, which are more resistant to coalescence and phase separation when compared to conventional emulsions. The droplet size is directly related to the color of nanoemulsions, which can vary from transparent to slightly cloudy [13,14]. Essential oil nanoemulsions applied in systems to extend food shelf life might improve the functionality of EOs by increasing the oil droplet surface area and, consequently, the contact area between the active agents and the food, enabling the use of smaller doses of essential oils [13,15]. In addition, oil droplets on the nanoscale are capable of easily diffusing into the microorganisms’ cellular membrane and disrupting its organization, promoting cellular internal content leakage and cellular death, which considerably improves antimicrobial capacity.

The use of essential oil nanoemulsions in active films and coatings is a promising alternative to increase the shelf life of anthracnose-susceptible tropical fruits. The performance of different essential oil emulsions in reducing deterioration levels depends on the chemical composition and the emulsion properties. To the best of our knowledge, this is the first review to focus on current trends related to the production of emulsions, especially nanoemulsions, involved in the development of active films and coatings for the preservation of tropical and subtropical fruits against the effects of infection by the genus *Colletotrichum*. In this context, three main topics are investigated: (a) the characteristics of the fungus and the disease that can lead to tropical fruit degradation during storage and, consequently, to significant economic losses; (b) the characteristics of essential oils and their mechanism of action that makes them potential natural food additives for fruits; (c) the use of conventional and nanoemulsions in edible films/coatings for inhibition of fungal growth and consequent prevention of anthracnose symptoms.

## 2. Anthracnose in Tropical Fruits

### 2.1. Economic Data

Tropical and subtropical fruits are widely consumed worldwide; therefore, the quality and diversity of these fruits must be preserved. The three main tropical fruit-producing countries are China, India, and Brazil. Brazil, despite being responsible for the third largest fruit production in the world, has a small representation in world fruit export levels. Only approximately three percent of its fruit production was exported in 2021. The failure to strengthen international agreements that have strict standards of commercialization is the main factor contributing to the low exportation levels. Commercial fruit growing increasingly requires professionalism and specific regulation, and fungal contamination can directly affect this commercial system. The low exported volume is related, among other causes, to the quality loss during storage and transport, which may result in the nonacceptance of fruits by the importing countries. Many of these tropical and subtropical fruits are susceptible to contamination by species of *Colletotrichum* [16,17].

Fruit losses are related to the decrease in the amount of food available for human consumption in the production, post-harvest, storage, and transport phases. According to the FAO [18], 14% of the world’s food is lost after harvesting and before reaching retail. Udayanga et al. [19] reported that a large part of the fruits grown in countries in the Asian region are lost due to improper handling, inefficient transport, and fungal and bacterial contamination. The trade and export of these fruits depend on the compliance with strict phytosanitary standards imposed by developed countries, which has an impact on agricultural trade in developing countries (major producers of tropical fruits). Anthracnose is a disease capable of causing economic impacts; therefore, technological investments are important to prevent the damage caused by this disease.

### 2.2. The Infectious Process of Tropical Fruits by Colletotrichum

The contamination process of healthy fruits is favored by the environmental factors of tropical regions. The causes are diverse and vary according to the region and the type of production. Generally, high temperatures (around 27 °C) and moisture (around 80%) highly affect anthracnose development in tropical fruits. Prolonged periods of rain in tropical regions together with high temperatures favor the progression of the disease. Constant moisture on the leaf surface stimulates the infectious process and fungal growth. In the case of fruits, most are lost due to contamination during the production and ripening process [17]. The control of environmental factors is an important point to be considered in the post-harvest and storage process. Post-harvest diseases are a major cause of fruit and vegetable losses in the world. In this sense, anthracnose becomes a major concern for producers because the fungus associated with this disease has caused losses in many species of mango, papaya, avocado, banana, cashew, carambola, guava, passion fruit, and other fruits [17,20].

**Figure 1:**
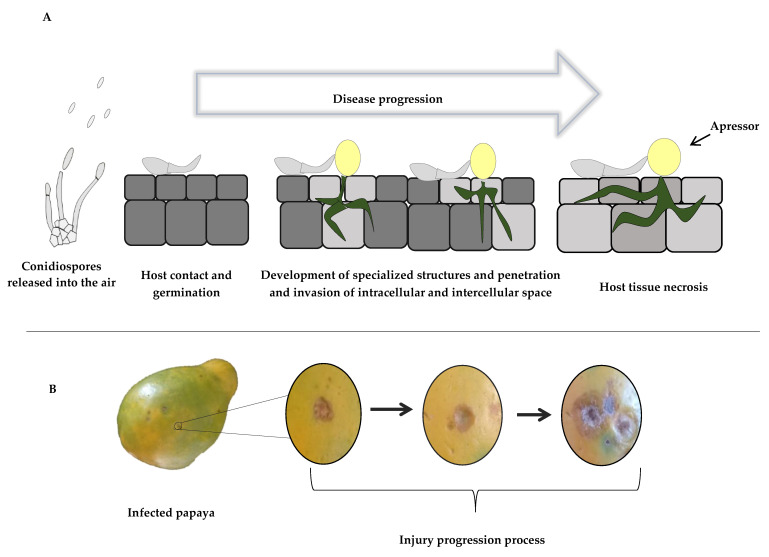
Scheme of microscopic (**A**) and macroscopic (**B**) view of anthracnose disease development.

Anthracnose is a disease characterized by the appearance of symptoms mainly during fruit ripening, i.e., during the post-harvest period, in climacteric species. Dead leaves and infected branches or fruits can be a source of contamination, and infection can occur at any stage between fruiting and harvesting. Conidiospores, fungal germ structures, can be dispersed by wind or water. When dispersed, they can adhere to the surface of the fruits and germinate in a short time. Soon after, they produce the germinal tube, which penetrates the cuticle of the fruit. After penetration, the hyphae can colonize the fruit wall. The initial epidermal lesions are small and circular, with a dark brown or, in some cases, black color. Small circular lesions become soaked with water, looking deeper than the surface of the fruit. They may increase in size with age, and the center of an older spot becomes blackened and develops gelatinous masses of pink or orange spores. In a few days, with the increase in size during ripening, the lesions present initial points of necrosis, leading to fruit loss (Figure 1) [19,21].

### 2.3. The Colletotrichum Lifestyle and Its Relationship with the Symptoms of Anthracnose

The relationship between fungi and plants is highly complex, as it depends on changes related to different stages of plant life, as well as on the physiological development, resistance to the host, the environment, and genes associated with the disease [22]. The *Colletotrichum* genus is formed by a group of important phytopathogens that cause large losses of fruits, vegetables, cereals, grasses, and ornamental plants in tropical regions [19]. There is no established standard for the lifestyle of the different species and groups that make up the *Colletotrichum* genus, hampering the control of the diseases caused by them [23].

The lifestyle of this genus can be broadly categorized by some characteristics that arise throughout the life cycle of the fungus. The *Colletotrichum* genus can be classified as follows: (i) necrophytes, almost all of which use lytic enzymes or toxins to cause cell death at some stage of the infectious process; (ii) endophytes, which inhabit plant cells in the process of symbiosis without causing apparent disease; (iii) quiescent species, whose fungus remains in latency or a dormant stage with no activity and generally becomes active in the post-harvest moment; (iv) biotrophic species, a characteristic common in the early lifestyle of the genus *Colletotrichum*, when the fungus remains alive inside the cells, actively absorbing the plant’s metabolites for its development without killing the cells [24,25].

The complex lifestyle of the *Colletotrichum* genus associated with its ability to change style and the potential to infect different host species are factors that hinder the management of contaminated fruits and vegetables. The lifestyle of this genus is highly regulated by specific genes and by specific biochemical interactions, in which enzyme activity and the production of secondary metabolites specific to the pathogen–host interaction occur [25]. In this process, it is necessary to consider not only symptomatic fruits but also asymptomatic ones, as both can disperse the infectious agent to other plants. Knowledge of the lifestyle, the different stages of development, and the mechanisms that species of *Colletotrichum* use for the disease progression is essential to avoid commercial losses and allow for the exportation of fruits and vegetables.

The structure of the host plant is an important point in the pre-infection moment, since the presence of cuticles, stomata, and trichomes and the thickness of the epidermis can be initial barriers to the infection process [26,27]. Generally, *Colletotrichum* infections start with the germination of conidia and the formation of specialized infectious structures (appressoria) that facilitate the entry through the cuticle and cell wall [28]. However, the species *C. gloeosporioides,* when infecting blackberry leaves, for example, formed specialized vesicles on or inside stomata, allowing the hyphae to enter the leaves [29]. In another case, *C. orbiculare* produced an appressorium to disrupt the plant’s surface and cause lytic enzymes to digest the cuticle and cell wall, allowing the conidia of this species to adhere [30].

After penetration, different species can start an intracellular or subcuticular hemibiotrophic process, generating an asymptomatic biotrophic phase without cell death. Subsequently, fungi can enter the necrotic phase, where secondary hyphae grow in the intracellular and intercellular space, which secrete degrading enzymes from the wall, leading to cell death [23]. In addition to this mechanism, some species may initiate the process of subcutaneous intramural necrotrophic infection, where fungi grow under the cuticle between the periclinal and anticline walls without the penetration of protoplasts [28,31]. Infections caused by *C. gloeosporioides* have already been reported as intracellular hemibiotrophic in guava fruits, where vesicles and hyphae of infection were formed in the initially infected epidermal cell [32].

The interaction between the host and the microbiological agent during the development of anthracnose is complex and dynamic. The different lifestyles that this genus can adopt make the effective and economical treatment of this disease in tropical and subtropical fruits even more difficult [25]. Losses from contamination can impact the entire production process, leading to economic damage [33]. The control and management risks associated with the infection by *Colletotrichum* are fundamental points to avoid the dispersion of the fungus and contamination of other fruits.

## 3. Conventional Treatments for Anthracnose Prevention

Tropical and subtropical fruits have been produced for subsistence and local distribution for many years. Investments in technology and improvements in transport and storage conditions have made the global commercialization of these fruits possible. The Southern Hemisphere has undergone significant changes in its production processes to reach the ideal standard for fruit exports to the Northern Hemisphere [34]. However, some challenges such as the lack of technology and infrastructure, high insect infestations, undesirable microbial growth, the appearance of lesions and stains due to improper handling or transport, and the prevalence of high temperatures and humidity still make the commercialization process difficult. In this context, the prevention of post-harvest diseases is essential to maintain the fruit trade and to reduce losses.

### 3.1. Physical Treatments

During the post-harvest stage, some traditional physical and chemical treatments can be applied to control the symptoms of anthracnose. Alvindia and Acda [35] evaluated the effects of hot water application on anthracnose-causing mango crops. In this study, treatment at 53 °C for 20 min had a significant effect on reducing the germination of *Colletotrichum gloeosporioides* spores after 48 h, preserving the fruit quality. However, the high amount of water used is a disadvantage of this type of treatment.

Other physical treatments have been evaluated in recent studies. Fischer et al. [36] used UV-C radiation to control anthracnose symptoms of and stem rot in avocado fruits inoculated with *C. gloeosporioides*. The applied doses of UV-C radiation (0.34–0.72 kJ·m^−2^) were not effective in reducing the occurrence of the disease. Contrarily, by applying a higher dose of radiation (3.0 and 4.0 kJ·m^−2^), Wanasinghe and Damunupola [37] successfully suppressed anthracnose symptoms on tomatoes. Nonetheless, these results should be evaluated with caution, because the authors observed the presence of antifungal compounds in tomato skins. Furthermore, radiation is still seen as a problem for consumers. Although it has minimal ambient impact, the lack of information about possible changes in food and, consequently, the possible impacts of this type of treatment on human health remain the main barriers to this method’s acceptance [38].

### 3.2. Chemical Treatments

Some chemical treatments are applied to reduce the symptoms of anthracnose in fruits, but most are toxic or less efficient over time as the pathogen’s resistance increases. Vieira et al. [39] evaluated the efficacy of thiophanate-methyl, a fungicide frequently used to control Black Sigatoka, on the growth of *Colletotrichum musae*, which causes anthracnose in bananas. Their results suggested a potential resistance of *Colletotrichum musae* to thiophanate-methyl, leading to treatment inefficacy. Thiabendazole showed poor growth control of *Colletotrichum gloeosporioides* in papaya, whereas the fungicides imazalil, prochloraz, propiconazole, and tebuconazole showed a positive effect by interrupting the germination of *Colletotrichum gloeosporioides* spores when applied at 50 ppm [40]. Even with satisfactory results, the repeated and continued use of these pesticides can change the balance of ecosystems, increase the incidence and severity of diseases, and still select isolates resistant to these chemical compounds [41].

A successful treatment for anthracnose control must consider environmental health impacts. Resources must be selected to maintain their balance in the environment, avoiding waste, without causing pollution or future damage. The treatments available to control anthracnose symptoms still face barriers, which can make them unfeasible or ineffective. The use of chemical or physical agents to reduce the growth of the *Colletotrichum* genus must meet safety criteria and environmental preservation. Therefore, an increasing number of studies have investigated potential natural pesticides capable of assuring fruit integrity, as well as safety and a reduction in losses.

## 4. Essential Oils

### 4.1. General Characteristics and Potential Applications

Several chemical substances have been applied to reduce the effects caused by fruit contamination after harvesting. Lately, most food producers have been forced to change this approach to meet the growing demand for chemical-free products, as the use of sustainable and safe natural products is progressively being demanded. Therefore, to meet consumer criteria, industries have had to look for new sources of compounds with effective action in the preservation process.

EOs are secondary plant metabolites that consist of a complex mixture of compounds extracted from various parts of plants, such as leaves, flowers, buds, seeds, branches, bark, herbs, wood, fruits, and roots, and they have been increasingly explored due to their insecticide, antioxidant, anti-inflammatory, antiallergic, and anticancer potential [42,43]. The bioactivity of the molecules present in EOs makes them very attractive to the food industry, both for direct application, as a potential natural preservative in food formulation, and for indirect application, in the production of active packaging, e.g., to improve food preservation by preventing pathogenic and/or spoilage microorganisms [44,45].

Carotenoids, alkaloids, phenolic compounds, flavonoids, isoflavonoids, and aldehydes are the groups commonly found in the composition of EOs [43]. Moreover, EOs are rich in substances classified as terpenes, terpenoids, and phenylpropanoid homologs [46], which contain several compounds associated with different bioactivities. Some studies have suggested that the antimicrobial activity of essential oils may be directly related to major components, but there is also a possibility of synergy or antagonism among the different components [47]. For example, Hyldgaard, Mygind, and Meyer [48] described the low efficiency of isolated terpenes against the growth of *Escherichia coli*, *Staphylococcus aureus*, *Bacillus cereus*, and the fungus *Saccharomyces cerevisiae*, and specific terpenoids such as carvacrol and thymol have been reported as potent antimicrobial agents when isolated. Chavan and Tupe [49] demonstrated the synergism between carvacrol and thymol in vitro and in vivo, which promoted membrane damage and cytoplasmic leakage of wine spoilage yeasts.

The characteristics of different essential oils can be exploited to prevent or reduce the damage caused by anthracnose in fruits. However, for the treatment to be effective, it is necessary to elucidate the mechanisms of action of the different components of EOs against fungal growth.

### 4.2. Mechanism of Action against Fungi

The mechanism of action of essential oils against fungal development is not yet fully understood. Nevertheless, some studies have suggested potential antimicrobial activity based on the functional group structures of compounds present in EOs [50]. The structure of compounds in EOs determines their hydrophobicity, allowing passage through the cell wall and membrane, leading to increased permeability and, consequently, to cell death or inhibition of sporulation and germination of fungi [51]. The literature reports that phenolic compound structures are associated with the high antimicrobial potential of clove, thyme, oregano, cinnamon, rosemary, sage, and vanilla EOs [47].

Hydrophobic components can interfere with synthesis reactions in wall structures, affecting morphogenesis and hyphal growth [52]. Some studies have suggested that hydrophobic compounds interact with ergosterol, the essential molecule that maintains the cellular integrity, viability, function, and normal growth of the fungus. Clove and thyme essential oils are effective in inhibiting ergosterol synthesis [53,54]. Changing the fluidity and permeability of the membrane leads to loss of ions, collapse of the proton pump, and reduction in membrane potential; moreover, in some cases, interactions between phenolic compounds and membrane proteins can occur, precipitating them and resulting in leakage of intracellular components [55,56].

The antifungal activity of essential oils may also be related to the disruption of fungal mitochondria. Some EOs can inhibit specific enzymes, such as mitochondrial ATPase, malate dehydrogenase, and succinate dehydrogenase, decreasing energy metabolism [57]. In addition, essential oils can alter the mitochondrial membrane of fungi, changing electron fluxes through the electron transport chain and, thus, producing altered levels of reactive oxygen species (ROS), which can oxidize and damage important molecules such as DNA, proteins, and lipids [58]. The increase in ROS is closely related to the biochemical process of cell death. Terpenes, for example, are structures capable of causing an increase in ROS species [54].

Some studies have shown promising results using essential oils to inhibit fungal growth. Essential oils of oregano, onion, mint, basil, and rosemary were tested against *Fusarium* sp., *Aspergillus ochraceus*, *Aspergillus flavus*, and *Aspergillus niger*. Oregano essential oil showed a fungicidal and fungistatic effect on all samples of fungi, while the other oils had a less pronounced effect, which could be improved by adjusting the dosage [59]. *Colletotrichum musae* and *Colletotrichum gloeosporioides* isolated from mango and banana fruits were treated with eugenol and rosemary (*Rosmarinus officinalis*), eucalyptus (*Eucalyptus citriodora*), and copaiba (*Copaifera langsdorffii*) essential oils. Rosemary and eucalyptus essential oils inhibited the growth of *C. musae*, copaiba oil was efficient against *C. gloeosporioides*, and eugenol showed antifungal activity against both species [60]. Some studies related the antifungal activity of secondary plant metabolites to their penetration into the hyphal wall, damaging the lipoproteins of the cytoplasmic membrane and leading to cytoplasmic extravasation, as well as emptying, dehydration of hyphae, and the presence of filaments [60,61].

The information presented in this section demonstrates the possible application of essential oils as biofungicides. The complex chemical profile and specific characteristics of the molecules present in EOs enable their antimicrobial effect. Although further elucidation regarding the mechanism of action against the *Colletotrichum* genus should be established, there is a robust body of data indicating the fungicidal properties of EOs. The versatility of essential oils, as well as the chemical variety present in these oils, can be an alternative to reduce the barriers imposed by the complex lifestyle of the genus *Colletotrichum*.

## 5. Active Films and Coatings

Films and coatings are thin layers, usually up to 0.3 mm thick, that have been used for centuries to protect foods from structural damage and nutritional losses [62]. Films and coatings can improve the physical strength of foods and can act as a barrier against gases and water vapor, decrease moisture migration, control microbial growth, reduce changes caused by light and oxygen, and improve visual and tactile characteristics. Thus, they are usually applied to increase the quality and shelf life of food products, protecting them from physical, chemical, and biological deterioration [63,64].

Biobased polymers commonly used in film and coating formulations are extracted from plants, animals, or microorganisms. Hydrocolloids are easily applied as biopolymers, with proteins and polysaccharides being the most used [65,66]. Some formulations can be added with oils or fats, such as triglycerides, waxes, free fatty acids, and vegetable oils, to improve the water vapor barrier properties, due to the hydrophobic nature of these materials. Biopolymer-based coatings have been applied to fruits such as mango, apricot, and papaya, promoting an increase in their shelf life by preserving texture and reducing weight loss and respiration rate [67,68,69].

The incorporation of substances with antimicrobial potential in the production of coatings and films has been suggested as a strategy to maximize their benefits to the quality and safety of fresh products and further increase their shelf life [70,71]. These are the so-called “active films and coatings”, which can promote the controlled release of active compounds. Depending on the application, migration rates can be reduced, immediate, extended, and specific or absent. Chemical bonds between the materials and the active substance must be considered, as well as the environmental factors that may regulate the migration process of the active compound. Many active compounds of different natures can be applied to form the active packaging, the most common being fertilizers, repellents, pesticides, antimicrobials, antioxidants, bioactive nutraceuticals, paints, and flavors [53,71].

As previously mentioned, EOs are substances with a broad spectrum of activity against fungi and bacteria, which can be added to films and coatings to increase the shelf life of foods [72]. EOs are highly volatile, unstable, and hydrophobic; therefore, they are usually added in the form of emulsions, ensuring uniform dispersion [73].

The use of coatings and active films containing essential oil emulsions to control phytopathogens has been explored by several researchers [74,75]. Cassava starch films incorporated with clove essential oil were applied to bananas and showed efficient antifungal activity against *Colletotrichum gloeosporioides* and *Colletotrichum musae* [76]. Most studies showed promising results, encouraging different research groups to continue improving the methods and techniques related to the formulation of these packages. An analysis of these studies will make it possible to understand the current results while presenting new alternatives for even more auspicious results. Although the future seems promising for this technology, the lack of machinery to produce films and coatings with high productivity and low energy consumption is a major challenge for scaling up this technology. Additionally, the mechanical strength and barrier properties of active packings need improvements to compete with the long-established petroleum-derived plastics; furthermore, as a new technology, films and coatings should prove their safety to conquer consumer acceptance [62].

## 6. Emulsions

Despite all their benefits, the use of EOs is a challenge, due to their lipophilic, volatile, and highly oxidizable nature [77]. These characteristics hamper the direct application of essential oils in foods, reducing their biological activity and promoting undesirable organoleptic alterations. A delivery system compatible with food applications can minimize these impacts and retain the biological activity of EOs [78]. In this sense, emulsions are easy-to-formulate delivery systems that can be applied to protect EOs against environmental factors and to ensure the effectiveness of their application.

### 6.1. Conventional Emulsions

An emulsion consists of two immiscible liquids, with one of them dispersed as small spherical droplets in the other. Emulsions formed by water and oil and can mainly be classified as water-in-oil (w/o) emulsions (water droplets dispersed in an oil phase) or oil-in-water emulsions (oil droplets dispersed in a water phase). They have relevant use in several areas, including the food sector. In addition to being present in many natural or processed foods, they are also used in delivery systems for functional compounds such as vitamins, nutraceuticals, aroma, flavor, color, and preservatives [13]. Emulsion systems enable the administration of active compounds through encapsulation, which can preserve and control the release of functional ingredients, improving efficiency, handling, and/or stability [79].

Some studies have incorporated EO emulsions in active coatings and films, exploring their antimicrobial activity to prevent anthracnose symptoms in different fruits. Emulsified essential oils from *Allium sativum*, *Copaifera langsdorfii*, *Cinnamomum zeylanicum,* and *Eugenia caryophyllata* were incorporated into polymeric coatings to control the symptoms caused by *Colletotrichum musae* in bananas. All treatments reduced the incidence, lesions, and disease severity [80]. The progression of anthracnose in papayas caused by *Colletotrichum gloeosporioides* and *Colletotrichum brevisporum* was prevented by applying chitosan coatings added with essential oil of *Mentha piperita L.* or *Mentha × villosa* Huds emulsions. The formulations containing 5 mg/mL of chitosan and 0.6 μL/mL of *Mentha piperita* oil or 1.6 μL/mL of *Mentha × villosa* Huds oil reduced the development of lesions similarly or superiorly to commercial fungicides [81]. The in vitro and in vivo potential of cassava starch films incorporated with lemongrass, thyme, and oregano essential oils was evaluated against *Colletotrichum musae* from bananas. Oregano oil inhibited mycelial growth and the complete in vitro germination of *C. musae* conidia. Lemongrass and thyme essential oils and two percent and three percent cassava starch films, as well as their combination, were effective in reducing and preventing anthracnose lesions in banana fruits [82]. All these studies suggest that the use of emulsified essential oils incorporated into coatings or films can inhibit the growth of anthracnose-causing fungus in tropical fruits.

Lately, several studies have shown that the droplet size strongly affects the bioactivity of essential oil emulsions. A nanoemulsion is a scarcely explored strategy in combating the *Colletotrichum* genus, but recent studies have indicated that its benefits can surpass those of macroemulsions, depending on the materials used and the application. On a nanometric scale, emulsions may have higher bioactivity and greater stability, and they may cause little change in the physicochemical characteristics of fruits [78,83].

### 6.2. Nanoemulsions

Conventional emulsions are characterized by an average droplet radius ranging between 100 nm and 100 µm, and nanoemulsions have an average droplet radius ranging between 10 and 100 nm [84]. Reducing droplets to a nanometric scale decreases the attractive forces acting on the droplets, consequently avoiding aggregation and coalescence. Furthermore, the conditions of physical stability are governed by Brownian motion, which ends up dominating the gravitational forces. As a result of the different forces acting on the droplets, nanoemulsions are more stable than emulsions [10,13,78].

Nanoemulsions are dispersions created with the aid of an energy source, which come from methods classified as high- or low-energy [85]. Low-energy methods are characterized by the spontaneous formation of small oil droplets, changing the solutions or the environmental conditions in which they are inserted. Phase inversion temperature, phase inversion composition, membrane emulsion, spontaneous emulsification, and solvent displacement/evaporation are some of the methods for producing nanoemulsions under low-energy conditions. These methods have some disadvantages, such as the use of large amounts of solvent [86] or synthetic surfactants [87] and difficulties in operating with large volumes of nanoemulsion solution [88].

High-energy methods use mechanical devices to create intense disruptive forces to disrupt the oil and water phases, forming tiny droplets. Methods such as rotor–stator, ultrasound, and microfluidic homogenization or high-pressure valves are the most frequently applied to form nanoemulsions in high-energy systems. The disadvantages of these methods may be associated with long processing times, especially in high-pressure systems in which many cycles should be employed to form a homogeneous nanoemulsion, promoting lipid droplet coalescence and temperature increase during production [83]. High temperatures are critical for EOs during nanoemulsion formation processes, because the compounds present in these oils are volatile and sensitive to temperature increases. In this context, it is worth mentioning that when high-energy methods are applied, it is necessary to evaluate the retention of essential oil at the end of the process and, thus, to quantify the possible losses during the formation of the nanoemulsion.

### 6.3. Advantages of Using Nanoemulsions over Conventional Methods

As the droplet size decreases, the biological activity of the compounds encapsulated in the nanoemulsion system increases. This is because the transport of active molecules across cell membranes is performed more easily, and there is a greater relationship between surface and volume, impacting reactivity [10]. According to Donsí and Ferrari [78], the fusion of the small droplets of the nanoemulsions with the phospholipid bilayer of the microorganisms facilitates their access through the membrane surface, allowing their rupture and leading to cell death. Anwer et al. [89] observed that clove essential oil nanoemulsion showed greater antimicrobial activity against several microorganisms (*Bacillus subtilis*, *Staphyloccocus aureus*, *Proteus vulgaris*, *Pseudomonas aeruginosa*, and *Klebsiella pneumoniae*) when compared to pure essential oil. Pongsumpun, Iwamoto, and Siripatrawan [90] reported that cinnamon essential oil nanoemulsions showed greater activity against several fungi (*Aspergillus niger*, *Rhizopus arrhizus*, *Penicillium* sp., and *Colletotrichum gloeosporioides*) than the conventional emulsion, with inhibition halos more than twofold larger.

Nanoemulsions can exhibit an apolar phase and droplet size-dependent optical transparency. Systems with a droplet size below 40 nm usually form a transparent solution, whereas droplets in the range from 40 to 100 nm can result in turbid nanoemulsions (depending on the content of the nonpolar compound); lastly, emulsions with droplets above 100 nm are usually white due to significant multiple scattering [78,91]. Determining this relationship between droplet size and nanoemulsion color is critical, as color is an important attribute when applying films and coatings on foods [92].

Nanoemulsions have been used as encapsulation and delivery methods for bioactive compounds, as they are kinetically more stable than macroemulsions and less susceptible to coalescence, cream formation, flocculation, and sedimentation [93]. They can be applied to extend the shelf life of foods, improving the stability and solubility of the encapsulated active compound. Furthermore, in nanoemulsified systems, the active compound does not interact with environmental factors, and its release is controlled and prolonged [94,95].

Nanoemulsions can provide droplets in sizes up to 200 nm, facilitating the interaction between the active compound and the fungus membrane. In this process, the availability, retention, and preservation of essential oils are improved [73]. Furthermore, in nanoemulsions, EOs behave differently than in simple emulsions. Nanoemulsified EOs can have a sustained release, prolonging the time of action against microorganisms. The essential oil of *Grammosciadium pterocarpum* Bioss. was incorporated in the free form and nanoemulsified in films formed with whey protein isolate. Antioxidant and antimicrobial activity tests showed that films formed with nanoemulsions had significantly higher results than those formed with free essential oil, confirming the distinct retention and release pattern of nanoemulsified oils in films described previously. In this case, the film with the nanoemulsified EO continuously released its EO content for a longer period of time, and the film with free EO discharged its EO content acutely in the medium [96].

Another advantage of using nanoemulsions is that when applied to films, there is better maintenance of the original matrix arrangement of the films because of the droplets’ nanometric diameter [97]. The nanoemulsified cinnamon essential oil was incorporated into pullulan-based films. The small diameter and uniform size distribution of the droplets formed in the nanoemulsion were associated with an increased EO retention rate and improved microbial activity of the film [98]. These studies corroborated the potential to expand the shelf life and quality of tropical fruits through the application of embedded EO nanoemulsion films and coatings.

Recent researchers have developed active films and coatings incorporated with EO nanoemulsions to improve the shelf life of many foods [99,100]. A coating formed with sodium alginate and eugenol, carvacrol, and cinnamaldehyde nanoemulsion was applied to Nanfeng mandarins. The results of this study showed an inhibitory effect on *Penicillium digitatum* growth and better stability of the physical parameters in treated fruits compared to untreated ones [100]. A thymol nanoemulsion incorporated in films prepared with chitosan and quinoa protein showed inhibition of *Botrytis cinerea* growth in cherry tomatoes [101].

## 7. Current Scenario: Essential Oils in Active Film and Coating Formulations

Currently, society is showing great concern for the loss/waste of fruits and vegetables, as these are essential components in daily human diets around the world. Tropical fruits are highly perishable and suffer from diseases that cause significant losses in both production and marketing. Anthracnose is a very common disease that can be found in a wide variety of tropical fruits. The wrong management of anthracnose in some countries could promote a deleterious economic impact. Treating anthracnose and controlling postharvest symptoms can bring great improvements to the production of various fruits.

The current scenario of studies using EOs for the treatment of anthracnose reveals the concern of scientists in seeking clean and sustainable methods for this problem. More efficient and less aggressive treatments are needed at this time. The articles cited in Table 1 indicated promising results regarding in vitro and/or fruit application tests. Therefore, this review proposes a deeper investigation of the application of nanoemulsified EOs for the treatment of anthracnose. The perspectives for the future are encouraging, and the knowledge gaps described in this review should be elucidated in the near future by robust and well-structured research. In this section, a survey of the main gaps and methods applied so far for the treatment of anthracnose with EOs is carried out.

A compilation of the most relevant studies found since the early 2000s, focusing on the use of essential oils incorporated or not into films and coatings for the treatment of fruits with anthracnose, is presented in Table 1. From these data, it can be concluded that essential oils have a high potential to combat anthracnose symptoms in tropical fruits. All studies had in vitro tests reporting the antifungal effect of the oils against the *Colletotrichum* species tested. Furthermore, most studies used essential oils with direct application on the surface of the fruit or in the form of emulsions (without using high- or low-energy methods to produce them).

The studies shown in Table 1 used essential oils from different sources or major components of EOs, with fungicidal and/or fungistatic capacity. Studies using active films and coatings for the treatment of anthracnose still focused on direct applications, spraying, or simple emulsions of EOs. The application of nanotechnology was not considered in most (approximately 80%) of these articles. Among the articles that considered the use of nanotechnology for the treatment of anthracnose and that used nanoemulsified essential oils, only two applied a nanoemulsion in films or coatings. Neither of these two studies presented a sensory evaluation of the fruits. Data on the sensory analysis of tropical fruits that were subjected to post-harvest treatment with films and active coatings are still scarce, especially for the treatment of anthracnose. Although nanoemulsions require less use of EOs, they are rich in aromatic compounds that can provide significant changes in the original characteristics of the fruit, which may or may not be acceptable to consumers. Sensory evaluation is an effective tool to evaluate the influence of active packings on the sensory characteristics of tropical fruits, such as alterations in the taste, odor, and general appearance of the fruits.

When evaluating the studies for the treatment of anthracnose using nanoemulsified EOs presented in this review, some gaps were noticed. Did the application of nanotechnology to form emulsions with even smaller droplets have a significant effect on reducing fungal growth? Did the droplet size remain nanometric after the nanoemulsions were added to film and coating solutions? These questions are important to justify the use of nanotechnology as a viable alternative to the treatment of anthracnose. Of the eight articles (Table 1) that used nanoemulsified EOs, only one presented data on the antifungal effect of the nanoemulsion in relation to the crude emulsion [90]. This result could elucidate the cost–benefit assessment of the process, as it justifies the use of energy-generating mechanisms, which require greater investment, for nanoemulsion production. In addition, the evaluation of droplet size after coating or film production would guarantee the presence of EO nanoemulsions in the final and most important stages of active packing development (application and storage). In the two studies [102,103] that presented EO nanoemulsions embedded in film or coating, there was no evaluation of the droplet size after the production of film/coating-forming solutions.

The data presented in Table 1 demonstrate studies that achieved highly promising results regarding the inhibition of fungal growth and a consequent reduction in anthracnose symptoms. Regarding the in vivo results, the authors reported the inhibition of mycelial growth, the inhibition of fungal growth, and a reduction in lesions. Generally, the main effect in vitro was fungicidal, while that in vivo was fungistatic. Results in vivo indicated the high power of infection of the fungus and its resistance to the treatments tested. Some studies showed promising results with the use of EOs incorporated into active coatings to minimize the symptoms of anthracnose [81,82,104,105]. However, these studies did not use nanoemulsified EOs. Most of the articles that produced nanoemulsions (reported in Table 1) with the objective of inhibiting the growth of different species of the genus *Colletotrichum* presented only in vitro results. This demonstrates how much improvement is needed for this technology to reach the consumer market.

From the data summarized in Table 1, it can be observed that the use of an active coating/film has not yet been exhaustively evaluated. Films and coatings are made of natural and biodegradable products that can provide greater stability to the fruit during storage. The respiratory process of fruits continues after harvest, and films and coatings act as a physical barrier against gas and water vapor exchange, delaying the ripening and deterioration processes [97,106]. These factors, associated with the antifungal effect of EOs against phytopathogens, can guarantee an increase in shelf life, minimizing the symptoms of fruits infected with the genus *Colletotrichum*.

**Table 1:** Major studies showing the use of essential oils in different forms to inhibit the growth of *Colletotrichum* species since the early 2000s.

Essential Oil	Application of Essential Oil	Coating/Film	Species	Fruits	Main Results	Author
Copaiba (*Copaifera langsdorffii*), cloves (*Syzygium aromaticum*), eucalyptus (*Eucalyptus citriodora*), and rosemary (*Rosmarinus officinalis*)	Direct under the isolated strains	-	*C. musae; C. gloeosporioides*	Mango and banana	Inhibition of mycelial growth in vitro	[60]
Mint (*Mentha piperita* L. and *Mentha × villosa* Huds)	Emulsion	Chitosan coating	*C. gloeosporioides; C. brevisporum*	Papaya	Total inhibition of mycelial growth in vitro; total inhibition of lesion development in vivo	[81]
Garlic (*Allium sativum*), copaiba (*Copaifera langsdorfii*), clove (*Eugenia caryophyllata*), and cinnamon (*Cinnamomum zeylanicum*)	Emulsion	-	*C. musae*	Banana	Better results of biomass loss, fruit diameter, and length; lower incidence and severity of the disease	[80]
Thyme (*Thymus vulgaris* L.)	Direct application to the coating	Chitosan, gum arabic, and *Aloe vera* coatings	*C. gloeosporioides*	Avocado	Increased activity of antioxidant enzymes and consequent delay in cell degeneration caused by *C. gloeosporioides*; delay in the spread of the disease	[107]
Thyme (species not identified by the author)	Direct application to the coating	Chitosan coating	*C. gloeosporioides*	Avocado	The treated fruits were firmer and had a lower incidence of *C. gloeosporioides*	[108]
Mint (*Mentha piperita* L.)	Emulsion	Chitosan coating	*C. asianum, C. fructicola, C. tropicale, C. dianesei, and C. karstii*	Mango	Decreased mycelial growth and decreased severity of lesion to fruit	[109]
Clove (*Eugenia caryophyllata*), lemongrass (*Cymbopogon citratus*), thyme (*Thymus vulgaris*), cinnamon (*Cinnamomum zeylanicum*), and oregano (*Origanum vulgare*)	Direct application to the film	Cassava starch film	*C. musae*	Banana	Inhibition of mycelial growth and in vitro germination; reduction in and prevention of anthracnose lesions in fruits	[82]
Clove (*Caryophillus aromaticus* L.), almond (*Acrocomia aculeata*), and neem (*Azadiractha indica* A.Juss)	Spraying on fruits	-	*C. gloeosporioides*	Mango	Lower rates of fruit mass loss and lower incidence and progression of anthracnose	[110]
Lemongrass (species not shown) and cinnamon (*Cinnamomum zeylanicum*)	Direct application to the film	Gum arabic coating	*C. musae and C. gloeosporioides*	Banana and papaya	80% anthracnose control in bananas and 71% in papaya	[111]
Oregano (*Origanum vulgaris*) and lemongrass (*Cymbopogon*	Incorporated in sachets	-	*C. gloeosporides*	Mango	Control of fungal development in vitro	[112]
Lemongrass (*Cymbopogon citratus*), cinnamon (*Cinnamomum zeylanicum*), anise (*Pimpinella anisum*), rosemary (*Rosmarinus officinalis*), tea tree (*Melaleuca alternifolia*), and mint (*Mentha piperita*)	Direct application to the fruits	-	*C. gloeosporioides*	Papaya	Fungitoxic (in vitro) and fungistatic (in vivo) effect and lesion reduction	[8]
Clove (*Syzygium aromaticum*)	Direct application to the coating	Cassava starch coating	*C. gloeosporioides*	Guava	Increased shelf life, delayed color change, and slowed down the evolution of anthracnose	[113]
Lemongrass (*Cymbopogon citratus*)	Emulsion	Chitosan coating	*C. asianum; C. siamense; C. fructicola; C. karstii; C.tropicale*	Guava, mango, and papaya	Inhibition of fungal growth in vitro; partial and total inhibition of lesion development in fruits	[2]
Basil (*Ocimum basilicum*)	Emulsion	Beeswax coating	*C. gloeosporioides*	Mango	Control of anthracnose in fruits and absence of physicochemical alterations	[114]
Noni (*Morinda citrifolia* L.), lemongrass (*Cymbopogon citratus* DC Stapf), and mastruz (*Chenopodium ambrosioides*)	Direct application to the coating	Carnauba wax, gelatin, paraffin, and sunflower oil coatings	*C. gloeosporioides*	Papaya	Reduction in fungal growth rate and inhibition of conidia germination; reduction of at least 7% in fruit mass loss	[115]
Thymol and carvacrol	Direct application to the film	Starch film	*C. gloeosporioides*	Papaya and mango	Reduced incidence of anthracnose symptoms in treated fruits; in vitro fungicidal and in vivo fungistatic effects	[116]
Thyme (*Thymus vulgaris* L.)	Direct application to the fruits	-	*C. musae*	Banana	Delay in fruit ripening and increased shelf life; reduction of 37.2% of disease in fruits treated with the essential oil	[1]
Rue (*Ruta graveolens* L.)	Emulsion	Chitosan coating	*C. gloesporoides*	Papaya	Significant inhibition of *Colletotrichum gloeosporioides* growth in vitro and in situ; the organoleptic characteristics of the fruits were not affected	[117]
Cinnamon (*Cinnamomum cassia*)	Emulsion	Chitosan, sodium alginate, and carrageenan coating	*C. acutatum*	Strawberries	No significant differences between the control and treated samples in sensory evaluation	[118]
Thyme (*Thymus daenensis* Celak), lavender (*Lavandula angustifolia* Mill.), mint (*Mentha piperita* Willd.), savory *(Satureja khuzistanica* Jamzad.), and cinnamon (*Cinnamomum zeylanicum* Blume)	Spraying on fruits	-	*C. gloesporoides*	Papaya	Improved fruit firmness and reduced lesion diameter; reduced levels of fruit decomposition	[119]
Thyme (*Thymus vulgaris*) and Mexican lime (*Citrus aurantifolia*)	Emulsion	Mesquite gum candelilla wax and mineral oil coating	*C. gloeosporioides*	Papaya	Control of fungal growth; reduction in infection and disease severity indices	[120]
Lemon ironbark (*Eucalyptus staigeriana*), pepper rosemary (*Lippia sidoides*), and louro-cravo *(Pimenta pseudocaryophyllus*)	Emulsion	Carboxymethylcellulose coating	*C. acutatum*	Strawberries	Good fungal inhibition capacity (in vitro); morphological degradation of hyphae; reduced severity of anthracosis in fruits	[121]
Ginger (species not identified by the author)	Direct application to the coating	Gum arabic coating	*C. gloeosporioides*	Papaya	Maintenance of firmness, color, acid titration, and fruit ripening; inhibition of fungal development.	[122]
Thyme (*Thymus vulgaris* L.)	Emulsion	Chitosan coating	*C. gloeosporioides*	Mango	Inhibition of fungal growth in vitro; control of disease symptoms in vivo	[123]
Thymol and *R*-(−)-carvone	Direct application to the film	Poly(lactic acid) (PLA)-based polymer film	*C. gloeosporioides*	Avocado	Suppression of mycelial growth in vivo	[124]
Common rue (*Ruta graveolens*)	Emulsion	Chitosan coating	*C. gloeosporioides*	Guava	Reduced fungal growth and increased stability for at least 12 days; promising results for mass loss, maturation index, respiratory rate, color, firmness, and water activity; inhibition of lesion development	[125]
Cinnamon (*Cinnamomum zeylanicum or Cinnamomum verum*)	Direct under the isolated strains	-	*C. acutatum*	Kiwifruit	Reduction in fungal growth and spore germination	[9]
Yarrow (*Achillea millefolium*), *Ferula kuma,* and mint (*Mentha longifolia)*	Direct application to the fruits	-	*C. nymphaeae*	Strawberry	Reduction in fruit rot caused by the fungus	[126]
Thyme (*Thymus daenensis* Celak.), savory (*Satureja khuzistanica* Jamzad.), mint (*Mentha piperita* Willd.), cinnamon (*Cinnamomum zeylanicum* Blume.), and lavender (*Lavandula angustifolia* Mill.)	Emulsion sprayed on the fruits	-	*C. gleoporiodes*	Avocado	Reduction in disease severity	[127]
Lemon-scented ironbark (*Eucalyptus staigeriana*), rosemary pepper (*Lippia sidoides*), and louro-cravo (*Pimenta pseudocaryophyllu*)	Direct application to the coating	Carboxymethylcellulose coating	*C. gloeosporioides*	Papaya	Reduction in disease severity	[128]
Basil (*Ocimum basilicum* and *Ocimum gratissimum*)	Emulsion	-	*C. musae*	Banana	Inhibition of growth in situ and in vivo	[129]
Basil (*Ocimum basilicum*), cinnamon (*Cinnamomum zeylanicum*), and rosemary (*Rosmarinus officinalis*)	Spraying on fruits	-	*C. musae*	Banana	Reduction in mycelial growth in vitro; decreased incidence of anthracnose in fruits and increased shelf life	[130]
Thyme (*Thymus vulgaris*), cinnamon bark (*Cinnamomum zeylanicum* Blume), and clove bud (*Syzygium aromaticum*)	Direct application to the fruits	-	*C. acutatum*	Strawberry	Decreased development of the disease	[131]
Lemongrass *(Cymbopogon citratus* L.)	Nanocapsules constituted by essential oil and polylactic acid	-	*C. acutatum* and *C. gloeosporioides*	Apples	Reduction in rot lesions	[132]
Garlic (*Allium sativum* L.)	Direct application to the coating	*Aloe vera* coating	*C. musae*	Banana	Increased resistance against disease development	[133]
Pepper tree (*Schinus molle*)	Essential oil nanoparticles	Chitosan nanoparticles coating	*C. gloeosporioides*	Avocado	Efficiency against the establishment of the fungus in fruits	[134]
Ginger, plai, and fingerroot (species not identified by the author)	Emulsion	Hydroxypropyl methylcellulose coating	*C.gloeosporioides*	Mango	Inhibition of fungal growth and development	[135]
Tea tree (species not identified by the author)	Nanoemulsion	Sodium alginate film	*C. musae*	Banana	Inhibition of fungal growth and reduction in anthracnose symptoms in fruits	[100]
Black mustard (*Brassica nigra*)	Nanoemulsion	-	*C. musae* and *C. capsici*	-	Low inhibition of mycelial growth	[136]
D-Limonene	Nanoemulsion	-	*C. gloeosporioides*	-	Inhibition of mycelial growth	[137]
Cinnamon (*Cinnamomum zeylanicum*)	Nanoemulsion	-	*C. gloeosporioides*	-	Inhibition of fungal growth	[90]
Black pepper (*P. nigrum* L.), cinnamon (*C. zeylanicum* Blume), Jamaica pepper (*P. dioica* Lindl.), clove (*S. aromaticum* (L.) Merr), anise (*Illicium verum* Hook. f.), and nutmeg (*Myristica fragrans* Houtt)	Nanoemulsion	-	*C. gloeosporioides*	-	Inhibition of fungal growth	[138]
Ginger oil (species not identified by the author)	Nanoemulsion	Carnauba wax coating	*C. gloeosporioides*	Papaya	Inhibition of the mycelial zone and effective control upon fungal growth in fruits	[102]
Clove (*Syzygium aromaticum*)	Nanoemulsion	-	*C. gloeosporioides*	-	Inhibition of fungal growth	[139]

The potential benefits of treating anthracnose with nanoemulsified EOs incorporated into films or coatings are numerous. However, there are major challenges to overcome in order for this treatment to become an alternative to conventional anthracnose treatments. The choice of EOs considering their properties, the definition of the production method of nanoemulsions, and the selection of the best polymer for film and coating formation are still the main research questions in this area. This reveals the absence of robust studies involving the application of nanoemulsified EOs in films and coatings for the control of anthracnose symptoms in tropical fruits.

Despite the great potential of EOs and the noticeable improvement in their properties when they are in the form of nanoemulsions, further data is needed to confirm nanoemulsion superiority compared to crude emulsion and pure oil. Studies with consistent results on the reduction in anthracnose symptoms in tropical fruits, especially those that are superior to conventional treatments, will be important to justify investments in this area. The evaluation of the commercial viability of the treatment using films and coatings should be considered. Tropical fruits are generally produced on a large scale to meet local consumption and export demands. Therefore, it would be interesting that future studies replicate a pilot-scale film/coating production; this type of work using highly commercialized fruits should bring awareness regarding technology viability, reality of the productive market, and the consumption profile.

From this review, the importance of future studies focusing on improving the formulation of nanoemulsions, as well as their application in tropical fruits, is clear. Evaluations of fruit quality and sensory characteristics, as well as the reduction in anthracnose symptoms, are essential to explain the use of coating-forming solutions or films loaded with nanoemulsions. With the increase in consumption of essential oils around the world, the regulation of these oils has been carried out little by little in partnership between organizations and governments. This scenario favors the use of EOs for the treatment of anthracnose from a commercial and a food safety point of view. In this sense, future studies that focus on the development of EO nanoemulsions incorporated in films and coatings can be an attractive and innovative alternative for the tropical fruit market.

## 8. Conclusions

This review has demonstrated that essential oil-based active films or coatings have been successful in treating anthracnose in many tropical fruits. The inhibition of the growth of the *Colletotrichum* genus in vitro and in vivo was a noticeable reality in almost all studies that used the active film or coating as a treatment. Studies showed that the application of essential oils in the form of nanoemulsions improved their antifungal potential, increased their efficacy, and reduced the amount needed, which are advantageous attributes from the sensory and economic points of view. However, to date, only a few recent studies have applied nanoemulsions for the treatment of anthracnose. In this way, nanoemulsions represent a reality that can be further explored in the area of food preservation, especially for the treatment of anthracnose. The advantages presented in this review reinforce the potential of nanoemulsions to improve the results already obtained and generate unprecedented results in inhibiting fungal growth and disease development. However, further studies are still needed for a cost–benefit analysis of this technology and to confirm the superiority and viability of EOs nanoemulsions incorporated with films and coatings compared to conventional treatments.

## Data Availability

Not Applicable.
